# Diet and development among children aged 36–59 months in low-income countries

**DOI:** 10.1136/archdischild-2021-323218

**Published:** 2021-12-24

**Authors:** Lilia Bliznashka, Nandita Perumal, Aisha Yousafzai, Christopher Sudfeld

**Affiliations:** 1 Global Academy of Agriculture and Food Security, The University of Edinburgh, Edinburgh, UK; 2 Department of Global Health and Population, Harvard University T.H. Chan School of Public Health, Boston, Massachusetts, USA; 3 Department of Nutrition, Harvard University T.H. Chan School of Public Health, Boston, Massachusetts, USA

**Keywords:** child development, child health, global health

## Abstract

**Objective:**

To assess the associations between diet, stimulation and development among children 36–59 months of age in low-income and middle-income countries (LMICs).

**Design:**

We pooled Demographic and Health Survey data on 12 126 children aged 36–59 months from 15 LMICs. Child diet indicators included dietary diversity score (DDS, range 0–7), minimum dietary diversity (MDD, defined as DDS ≥4) and animal source foods (ASFs) consumption. Child development was assessed using the Early Childhood Development Index and stimulation by the number of stimulation activities (range 0–6). Associations were assessed using generalised linear models.

**Results:**

In our sample, 18% of children met MDD and 50% received ≥4 stimulation activities. The prevalence of suboptimal cognitive, socioemotional, literacy-numeracy and physical development was 24%, 32%, 87% and 11%, respectively. Higher DDS, meeting MDD and consuming ASFs were associated with 8%–13% more stimulation activities. Children who met MDD were slightly less likely to have suboptimal literacy-numeracy development compared with children who did not meet MDD: relative risk 0.97 (95% CI 0.95 to 1.00). DDS, meeting MDD and ASFs consumption were not associated with cognitive, socioemotional or physical development. However, there was evidence of positive associations between MDD and cognitive and literacy-numeracy development among subgroups of children, including those who received ≥4 stimulation activities or attended an early childhood care and education programme.

**Conclusions:**

Child diet was associated with more stimulation activities. However, independent of stimulation, socioeconomic status and other factors, child diet appeared to be a prominent determinant only of literacy-numeracy development among children 36–59 months of age.

## Abstract (precis)

This detailed analysis of Demographic and Health Survey data from children in 15 LMICs demonstrates remarkable connections between diet and cognitive development.

**What is already known on this topic?**

- Adequate nutrition and opportunities for early learning are key components of nurturing care for child development in early life.
- Considerable literature has examined the associations between child nutrition and development in the first 2 years of life.
- Little is known about the role of nutrition in child development in children 36–59 months of age.

**What this study adds?**

- Dietary diversity was associated with literacy-numeracy development in children aged 36–59 months in low-income and middle-income countries, but not with cognitive, socioemotional or physical development.
- We found evidence of beneficial associations between child diet and development among subgroups of children: those who received ≥4 stimulation activities or attended preschool programmes.

## Introduction

In low-income and middle-income countries (LMICs), 25% of children 36–59 months of age have suboptimal development,^1 2^ which is associated with lower intelligence later in life.^3^ Adequate nutrition and opportunities for early learning are key components of nurturing care for early childhood development.^4–6^ Child nutrition may affect cognitive development directly through brain development and indirectly by affecting child health, physical activity and caregiver behaviour.^7–9^ Directly, deficiencies in protein and energy can affect global and motor function, whereas deficiencies in individual micronutrients (eg, iron, zinc) can affect specific cognitive processes and affective development.^9^ Indirectly, child diet can influence development by reducing activity, limiting exploration of the environment and reducing initiation of caregiver interactions.^8 10^ Caregivers who supply less diverse diets may supply less diverse stimulation.^11^ Conversely, caregivers who supply less diverse stimulation may supply less diverse diets. However, many factors influence child diet, stimulation and development. Therefore, these inter-relationships are important to consider.

Supplementation with individual (eg, iron, zinc) or multiple micronutrients has shown mixed or no effects on child development,^12 13 13–16^ while observational studies generally indicate that better-quality diets are associated with improved child development.^7 16–22^ These differential findings may be because supplementation trials usually consider single micronutrients and observational studies consider both macronutrients and micronutrients. However, most evidence comes from children aged <2 years. Little is known about the association between diet and development among children 36–59 months of age in LMICs. Similarly, a few studies have assessed the association between diet and stimulation in children aged <2 years,^16 23^ but evidence on children aged 36–59 months is lacking. Given this limited evidence, our objective was to understand diet as a risk factor for suboptimal development in children 36–59 months of age in LMICs, a critical period due to limited resources and interventional support (usually focused on the first 1000 days).

## Methods

### Study design

We pooled cross-sectional data from the latest Demographic and Health Surveys (DHS) for the 15 countries with data on child development, diet and stimulation among children 36–59 months of age that were publicly available as of December 2020 (online supplemental table 1). Child development, diet and stimulation for this age group are optional modules and available for a limited number of countries. Child development and stimulation are applied to the youngest child aged 36–59 months, and child diet to one randomly selected child in this age group. We excluded Multiple Indicator Cluster Surveys (MICS), which do not collect diet data for children aged 36–59 months.

10.1136/archdischild-2021-323218.supp1 Supplementary data

### Measures

Child diet was assessed using the WHO-UNICEF indicators for dietary diversity score (DDS) and minimum dietary diversity (MDD). DDS was created by summing the number of food groups consumed by the child in the past 24 hours (based on maternal recall). MDD was defined as DDS ≥4.^24^ We also created a binary indicator for whether the child consumed animal source foods (ASF, eggs/meat/flesh foods/fish/dairy).

Child development was assessed using the Early Childhood Development Index (ECDI) (additional details in online supplemental methods). The child’s mother reported on whether the child can perform 10 developmental milestones (table 1). Online supplemental table 2 shows mean age of children who can and cannot perform each milestone. We constructed indicators for whether children were developmentally on track in each domain and all four domains (overall development).^25^ Since we were interested in diet as a risk factor, our outcome was off-track development. We also calculated ECDI score as the number of milestones the child passed (range 0–10).

**Table 1:** Developmental milestones included in the Early Childhood Development Index by domain and coding of on-track and off-track development by domain

Domain	Milestone	On-track development if child	Off-track development if child
Cognitive	Follows simple directions on how to do something correctly	Passes ≥1 milestone	Fails both milestones
	When given something to do, is able to do it independently		
Socioemotional	Gets along well with other children	Passes ≥2 milestones	Fails ≥1 milestone
	Does not kick, bite or hit other children		
	Does not get distracted easily		
Physical	Can pick up a small object with two fingers, like a stick or a rock from the ground	Passes ≥1 milestone	Fails both milestones
	Is not sometimes too sick to play		
Literacy-numeracy	Can identify/name at least 10 letters of the alphabet	Passes ≥2 milestones	Fails ≥1 milestone
	Can read at least four simple, popular words		
	Knows the name and recognises the symbol of all numbers from 1 to 10		

Stimulation was assessed using the DHS home stimulation module. Mothers reported on whether any adult provided any of six stimulation activities in the past 3 days: reading books, telling stories, naming/counting/drawing, singing, taking the child outside and playing. We summed the total number of stimulation activities (range 0–6), and defined adequate stimulation as ≥4 activities, based on prior work from the MICS^26^ (additional details in online supplemental methods).

### Statistical analysis

We restricted the analytic sample to children 36–59 months of age with data on child diet, development and stimulation. DHS calculate child age as the difference between the interview data and date of birth (imputed if incomplete).^27^ We first examined the association between child diet and stimulation, treating stimulation as the outcome. Then, we examined the association between child diet and development, treating stimulation as a covariate. For binary outcomes, we fit log-Poisson models and calculated unadjusted and adjusted relative risks (RR) and 95% CIs. For count outcomes, we fit a linear model and calculated unadjusted and adjusted mean differences (MD) and 95% CIs. We calculated per cent increase by dividing the MD by the sample mean. Adjusted estimates controlled for household wealth, rurality, size, access to improved sanitation and access to improved water source^27^; maternal age, education and marital status; child age, sex and early childhood care and education programme (ECCE) attendance, and country and survey year. The models for child development also controlled for the number of stimulation activities. Missing data on any of the confounders (<0.10% of observations) was imputed using mean imputation. All models accounted for clustering and representativeness using the country-specific cluster variables and sampling weights. As a sensitivity analysis, we examined heterogeneity in the associations between child diet, stimulation and development between countries by fitting the multivariable adjusted model separately for each country. In the pooled sample, we also explored whether the multivariable adjusted associations between child MDD, ASF consumption and development differed across household wealth, rurality, household size, access to improved sanitation and access to improved water source; maternal age, education and marital status; adequate stimulation and child age, sex and ECCE attendance. The significance of the interaction was assessed using a Wald test. All analyses were performed in Stata V.16 and a p<0.05 was considered to be statistically significant.^28^

## Results

The analytic sample included 12 126 children 36–59 months of age (table 2). Child diet was poor with 18% meeting MDD. Half of children received adequate stimulation and 17% attended ECCE programmes. Child development was suboptimal: 24% of children were off-track in cognitive development, 32% in socioemotional and 87% in literacy-numeracy. Child development did not differ by age group: 35–47 vs 48–59 months (data not shown).

**Table 2:** Household, maternal and child characteristics of the 12 126 children in the analytic sample

	Mean (±SD, range) or proportion
Household characteristics	
Size	7.78 (±4.36, 3–56)
Lives in rural area	70.64
Is in poorest wealth quintile	26.00
Access to an improved water source	
Has access	30.38
Does not have access	66.44
Unknown	3.17
Has access to improved sanitation	29.28
Mother characteristics	
Age, years	29.35 (±5.88, 16–49)
Highest level of education	
No education	42.47
Primary education	33.96
Secondary or higher education	23.57
Married or cohabitating	95.73
Child characteristics	
Male	50.76
Age, months	47.12 (±6.79, 36–59)
Cognitive development off-track	23.75
Socioemotional development off-track	32.05
Literacy-numeracy development off-track	86.55
Physical development off-track	10.76
Overall development off-track	14.13
Early Childhood Development Index Score (0–10)	4.99 (±1.80, 0–10)
Child diet in the last 24 hours	
Consumed grains, white roots or tubers	54.95
Consumed legumes or nuts	23.00
Consumed eggs	12.87
Consumed flesh foods	32.69
Consumed dairy	11.99
Consumed vitamin A-rich fruits and vegetables	36.58
Consumed other fruits and vegetables	16.46
Dietary diversity score (0–7)	1.88 (±1.79, 0–7)
Met minimum dietary diversity (≥4 food groups)	18.18
Consumed animal source foods	38.32
Number of stimulation activities received in the past 3 days (range 0–6)	3.21 (±2.05, 0–6)
Received adequate stimulation in the past 3 days (≥4 activities)	49.67
Child attends an early childhood education programme	16.50

Child diet was positively associated with stimulation in unadjusted and multivariable models (table 3). In multivariable analyses, meeting MDD was associated with MD 0.42 (95% CI 0.31 to 0.53) or 13% additional stimulation activities, and ASF consumption with MD 0.25 (95% CI 0.16 to 0.33) or 8% additional stimulation activities. Results were generally consistent by country, although not significant in all countries (online supplemental tables 3–5).

**Table 3:** Associations between child diet and stimulation among children 36–59 months of age in 15 low-income and middle-income countries*

	Number of stimulation activities received		Adequate stimulation received	
	Unadjusted mean difference (95% CI)	Adjusted mean difference (95% CI)	Unadjusted relative risk (95% CI)	Adjusted relative risk (95% CI)
Dietary diversity score (0–7)	0.18 (0.15 to 0.20)	0.09 (0.07 to 0.12)	1.07 (1.06 to 1.08)	1.04 (1.02 to 1.05)
Minimum dietary diversity (≥4 food groups)	0.85 (0.72 to 0.97)	0.42 (0.31 to 0.53)	1.34 (1.27 to 1.41)	1.17 (1.11 to 1.23)
Consumed animal source foods	0.43 (0.33 to 0.53)	0.25 (0.16 to 0.33)	1.15 (1.10 to 1.20)	1.10 (1.05 to 1.15)

*All models applied country-specific cluster variables and sampling weights. Adjusted estimates controlled for household wealth, rurality, size, access to improved sanitation and access to improved water source; maternal age, education and marital status; child age, sex and attendance of an early childhood education programme and country and survey year.

Child DDS, meeting MDD and ASF consumption were not associated with overall, cognitive, socioemotional or physical development in multivariable models (table 4). However, higher DDS and meeting MDD were associated with lower likelihood of suboptimal literacy-numeracy development, but the magnitude of these associations was very small. These associations appeared to be largely driven by three countries: Congo, Timor-Leste and Uganda (online supplemental tables 6–8). In sensitivity analysis in the pooled sample, meeting MDD was associated with MD 0.12 (95% CI 0.01 to 0.23) higher ECDI score, whereas DDS and ASF consumption were not (online supplemental table 9).

**Table 4:** Associations between child diet and child development among children 36–59 months of age in 15 low-income and middle-income countries*

	Overall development off-track		Cognitive development off-track		Socioemotional development off-track		Literacy-numeracy development off-track		Physical development off-track	
	Unadjusted relative risk (95% CI)	Adjusted relative risk (95% CI)	Unadjusted relative risk (95% CI)	Adjusted relative risk (95% CI)	Unadjusted relative risk (95% CI)	Adjusted relative risk (95% CI)	Unadjusted relative risk (95% CI)	Adjusted relative risk (95% CI)	Unadjusted relative risk (95% CI)	Adjusted relative risk (95% CI)
Dietary diversity score (0–7)	0.92 (0.89 to 0.95)	0.98 (0.95 to 1.02)	0.92 (0.90 to 0.94)	0.99 (0.97 to 1.01)	1.01 (1.00 to 1.03)	1.02 (1.00 to 1.04)	0.97 (0.96 to 0.98)	0.99 (0.99 to 1.00)	0.94 (0.90 to 0.98)	0.99 (0.95 to 1.03)
Minimum dietary diversity (≥4 food groups)	0.70 (0.59 to 0.83)	0.92 (0.78 to 1.09)	0.65 (0.58 to 0.74)	0.91 (0.80 to 1.03)	1.02 (0.93 to 1.11)	1.04 (0.95 to 1.14)	0.86 (0.83 to 0.89)	0.97 (0.95 to 1.00)	0.88 (0.73 to 1.05)	0.98 (0.81 to 1.19)
Consumed animal source foods	0.8 (0.71 to 0.89)	0.97 (0.87 to 1.10)	0.79 (0.72 to 0.86)	1.01 (0.93 to 1.09)	1.04 (0.97 to 1.11)	1.05 (0.98 to 1.12)	0.93 (0.91 to 0.95)	0.99 (0.97 to 1.01)	0.94 (0.82 to 1.09)	1.02 (0.89 to 1.18)

*All models applied country-specific cluster variables and sampling weights. Adjusted estimates controlled for household wealth, rurality, size, access to improved sanitation and access to improved water source; maternal age, education and marital status; stimulation; child age, sex and attendance of an early childhood education programme and country and survey year.

**Figure 1:**
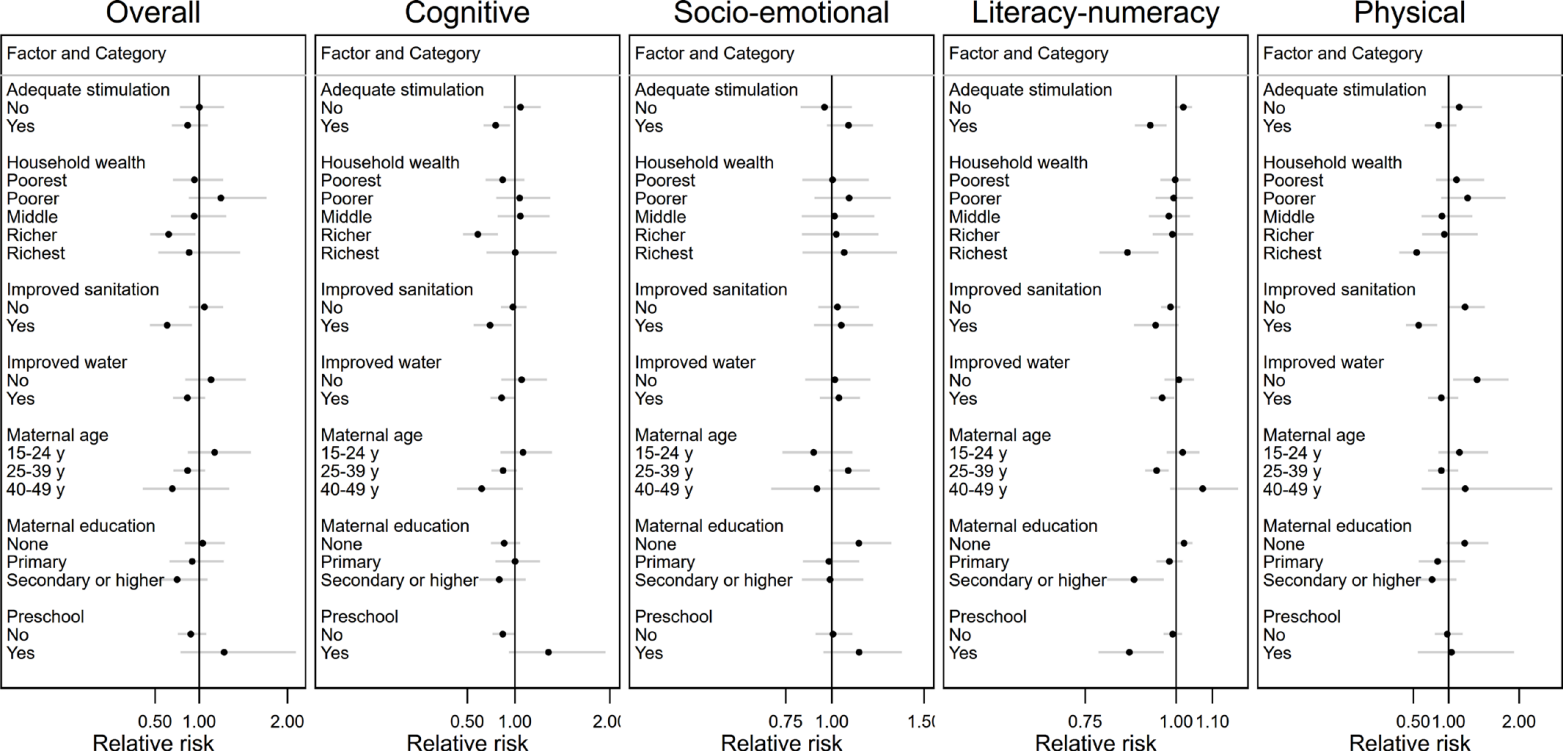
Heterogeneity of the association between child minimum dietary diversity and suboptimal child development by child, maternal and household factors, comparing children who met minimum dietary diversity and children who did not. Displayed are only factors that modified the associations with at least one child development domain, that is, p value for interaction was <0.05. All values are relative risk and 95% CIs. All models applied country-specific cluster variables and sampling weights. Estimates controlled for household wealth, rurality, household size, access to improved sanitation and access to improved water source; maternal age, education and marital status; stimulation; child age, sex and attendance of an early childhood education programme (preschool) and country and survey year.

In addition, we found that the magnitude of the associations between MDD and suboptimal cognitive and literacy-numeracy development was larger among children who received adequate stimulation compared with those who received inadequate stimulation (p values for interaction <0.05) (figure 1, online supplemental table 10). There was evidence of more beneficial associations among children with access to improved sanitation, older mothers, mothers with secondary or higher education and living in richer households (all p values for interaction <0.05). Lastly, ECCE attendance modified the association between MDD and cognitive development (p value for interaction 0.02) with a larger association among children not attending ECCE programmes, and the association between MDD and literacy-numeracy development (p value for interaction 0.02) with larger association among children attending ECCE programmes.

**Figure 2:**
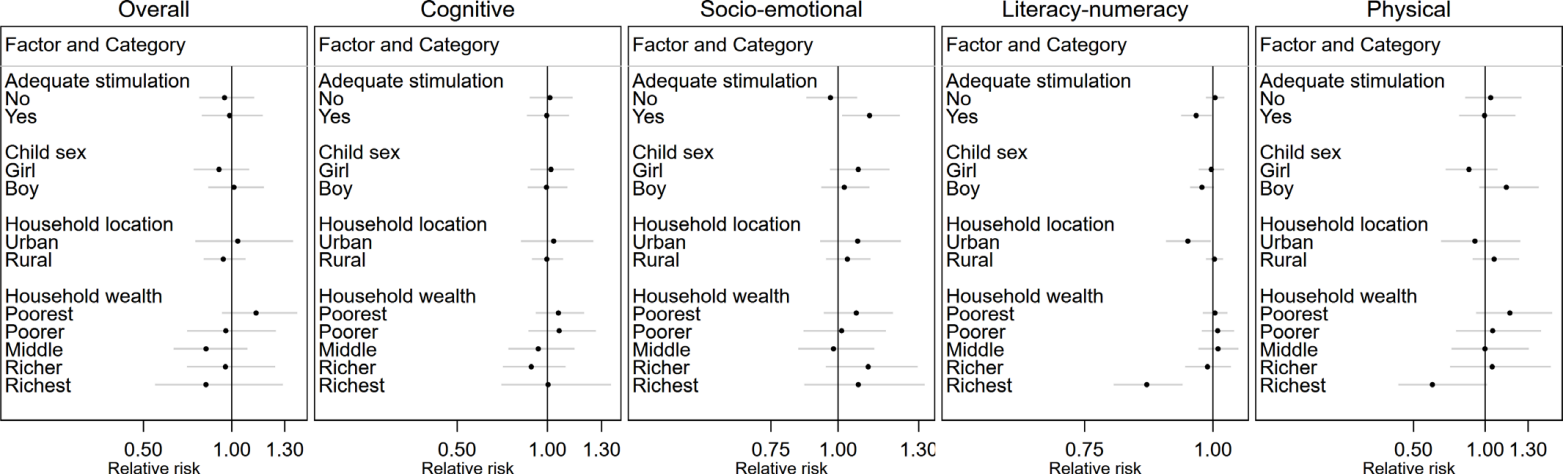
Heterogeneity of the association between child consumption of animal source foods and suboptimal child development by child, maternal and household factors, comparing children who consumed animal source foods and children who did not. Displayed are only factors that modified the associations with at least one child development domain, that is, p value for interaction was <0.05. All values are relative risk and 95% CIs. All models applied country-specific cluster variables and sampling weights. Estimates controlled for household wealth, rurality, household size, access to improved sanitation and access to improved water source; maternal age, education and marital status; stimulation; child age, sex and attendance of an early childhood education programme and country and survey year.

Likewise, adequate stimulation modified the association between ASF consumption and socioemotional and literacy-numeracy development with the magnitude of the association larger among children who received adequate stimulation (p values for interaction <0.05) (figure 2, online supplemental table 11). Additionally, household wealth modified the association between ASF consumption and literacy-numeracy development with more beneficial associations among children in wealthier compared with poorer households (p value for interaction 0.01).

## Discussion

We found that dietary diversity was positively associated with stimulation, literacy-numeracy development and ECDI score among children 36–59 months of age in LMICs, but not with cognitive, socioemotional or physical development. Child and household factors may modify the associations between child diet and development with beneficial associations for children receiving adequate stimulation, attending ECCE programmes and with access to improved sanitation.

Our findings that more diverse child diets were associated with more stimulation build on a limited literature suggesting similar associations in children aged <2 years.^16 29^ However, evidence suggests that patterns of association differ by setting, age group and season. Specifically, Wachs *et al* showed that, among children aged 18–30 months, Egyptian children who received more diverse diets received more diverse stimulation, whereas Kenyan children who received more diverse diets received less diverse stimulation.^29^ In rural India, stimulation mediated the relation between dietary diversity and mental development in children aged 12–18 months, but not in children aged 6–11 months.^16^ However, in a different sample of children aged 12–18 months living in the same area, assessed ~1.5 years later in winter, dietary diversity was not associated with development either directly or indirectly through stimulation.^23^ More research, including longitudinal studies, are needed to understand the relationships between child diet and stimulation, their heterogeneity and the mechanisms behind them.

Prior studies have shown that children with more diverse diets from 6 to 24 months of age have better development outcomes.^7 16 19–22^ Among (pre-)school-aged children, meta-analyses have assessed the association between diet quality and development,^17 18 30^ but only one included studies among children aged 36–59 months.^18^ A study in Scotland showed that more slow meals (ie, sit down meals; meals with fresh ingredients) were associated with better cognitive performance at ages 3 and 5.^31^ Additionally, a trial among Indian preschoolers 29–49 months of age showed that, compared with placebo, fortification of school meals with multiple micronutrient powders for 8 months improved expressive language, inhibitory control and socioemotional development in low-quality but not high-quality preschools. However, there were no effects on receptive language, fine motor development or visual reception.^32^ Our findings of limited associations between child diet and development build on this limited literature by providing evidence specific to children aged 36–59 months in LMICs. In this age range, children’s brains are no longer developing as rapidly as during pregnancy or earlier in life and nutrient requirements for ongoing brain development processes, such as higher cognitive function (eg, working memory, inhibition), are much smaller.^8 33^ Thus, in children aged 36–59 months, diet may no longer be as important of a predictor of the child development domains we assessed compared with earlier in life. Or it may be too homogenous to capture differences in child development.

The lack of associations between ASF consumption and child development in our study contrasts prior evidence showing ASFs benefit child development among primary school-aged children.^8 34–37^ However, we lacked data on ASF quantity or frequency of consumption. It is possible that neither was sufficient to show an association with child development or that ASF nutrients were prioritised towards other developmental or physiological needs.^38^

Our analysis of potential modifiers highlighted the role of adequate stimulation, ECCE programmes and improved sanitation. With respect to stimulation, we observed beneficial associations for cognitive and literacy-numeracy development among children with better diets, but poorer socioemotional development among children who consumed ASFs. The latter may be a chance finding given the number of potential modifiers explored. Additional research is needed to confirm this finding and clarify potential mechanisms. With respect to ECCE attendance, in a previous study, the effect of micronutrient fortification on child development in India was modified by preschool quality.^32^ We lacked data on ECCE quality; however, it was likely highly variable given that we included both urban and rural programmes in 15 countries. Nevertheless, ECCE programmes may influence child development by enhancing learning, identifying and treating learning and behavioural problems^39^ or serving as platforms for nutrition interventions. Lastly, improved sanitation likely reduces exposure to pathogens and environmental risks contributing to poor child development through persistent immune stimulation and poor gut health.^40^ Although promising, these findings on potential modifiers should be interpreted with caution, given the wide CIs for many of the subgroups we examined.

There are several important limitations of our study. First, we lacked data on macronutrient and micronutrient intake and only had data on food groups from a single 24-hour period. Prior studies among children aged <2 years suggest that the association between child diet and development may be prospective with better diet in early life predicting improved development in later infancy.^41 42^ Furthermore, the child diet indicators we used were developed to assess feeding in children aged <2 years and have only been validated for older children in Burkina Faso.^43^ Lastly, child diet depends on multiple socioecological factors (eg, food security, nutrition knowledge) not collected by DHS that may be important confounders or modifiers. Future research should consider these broader contextual factors in the associations between child diet, stimulation and development. Another limitation is the crude nature of the ECDI, which relies on 10 caregiver-reported items and is therefore limited in its ability to comprehensively assess each domain. Moreover, the ECDI does not assess higher cognitive functions (eg, attention, processing speed), which develop rapidly between 36 and 59 months of age.^33^ Child diet may be more important for these more rapidly developing domains as demonstrated by the positive effects of micronutrient fortification on inhibitory control in India.^32^ Furthermore, the literacy-numeracy domain has been criticised for containing more advanced items than comparable development assessment tools for children aged 36–59 months; the physical domain contains items that are less advanced than comparable tools.^44^ These limitations are evidenced in our sample where 87% of children had suboptimal literacy-numeracy development and only 11% had suboptimal physical development. Given these limitations, our results are hypothesis generating and should be interpreted with caution before being replicated using more comprehensive child development assessments. Last, our findings may not be generalisable to all LMICs given the small number of counties with child development, diet and stimulation data for children aged 36–59 months.

In conclusion, we showed that child diet was positively associated with stimulation and literacy-numeracy development among children aged 36–59 months in LMICs. Child diet was not associated with cognitive, socioemotional or physical development overall, but we found beneficial associations among children receiving adequate stimulation, attending ECCE programmes and with access to improved sanitation. Interventions that address child diet alone may provide limited benefits for child development from 36 to 59 months of age. Future interventions should consider holistic approaches to support child development in the second 1000 days that broadly address child diet, stimulation, ECCE access and other environmental factors.

## Data Availability

Data are available in a public, open access repository. The data underlying the results presented in the study are publicly available from the DHS Program (http://www.dhsprogram.com). Registration is required to access the data.
