# Supplementary material for: Diet and development among children aged 36–59 months in low-income countries
Source: Arch Dis Child. 2021 Dec 24;107(8):719–25. doi: 10.1136/archdischild-2021-323218 (PMC9304107; doi:10.1136/archdischild-2021-323218)
Supplement: Supplementary data [file archdischild-2021-323218supp001.pdf]

Supplemental Methods

Measures

Early Childhood Development Index (ECDI)

Early Childhood Development Index (ECDI) is a population-based measure to assess cognitive, socio-emotional, literacy-numeracy, and physical development among children 36-59 months of age (1). The physical domain covers gross motor development and illness. ECDI consists of 10 developmental milestones with the child's mother or primary caregiver reporting on whether the child can perform each of the 10 milestones. The 10 milestones were selected from an original list of 158 items based on test-rest reliability, inter-rater reliability, and predictive validity against existing child development tools through a multistage, multi-country validation process (2,3). Per the original ECDI coding guidelines, we constructed indicators for whether children were developmentally on-track in each domain and all four domains (overall development) (1). Since we were interested in diet as a risk factor, our outcome was off-track development. We also calculated a count ECDI score for the number of milestones the child passed (range 0-10).

Stimulation

Stimulation was assessed using the DHS home stimulation module, which collects data on whether adults provided any of the following six stimulation activities in the past three days (based on maternal report): reading books or looking at pictures, telling stories, naming/counting/drawing, singing, taking the child outside, and playing with the child. We summed the total number of stimulation activities (range 0-6) provided by any adult, and defined adequate stimulation as providing ≥4 stimulation activities, based on prior work from the MICS (4). This definition simultaneously ensures that children receiving adequate stimulation receive most of the six activities and that parents providing adequate stimulation are not penalized for being unable to

Bliznashka L, *et al. Arch Dis Child* 2022; 107:719–725. doi: 10.1136/archdischild-2021-323218

provide certain activities due to cultural or resource-related reasons (5). These six stimulation activities have shown acceptable short-term reliability and predictive validity against child cognitive, motor, and language development (6), and specifically against the ECDI (7,8).

1. Loizillon A, Petrowski N, Britto P, Cappa C. Development of the Early Childhood Development Index in MICS surveys. MICS Methodological Papers, No. 6, Data and Analytics Section, Division of Data, Research and Policy, UNICEF New York.; 2017.
2. UNICEF. The formative years: UNICEF's work on measuring early childhood development [Internet]. 2019. Available from: https://data.unicef.org/resources/the-formative-years-unicefs-work-on-measuring-ecd/
3. Janus M, Brinkman S, Duku E. Recommendations for the MICS ECD module. Second interim recommendations report part I. New York; 2008.
4. UNICEF. Inequalities in early childhood development: What the data say. New York, NY: United Nations; 2012.
5. McCoy DC, Salhi C, Yoshikawa H, Black M, Britto P, Fink G. Home- and center-based learning opportunities for preschoolers in low- and middle-income countries. Child Youth Serv Rev. 2018 May;88:44–56.
6. Hamadani JD, Tofail F, Hilaly A, Huda SN, Engle P, Grantham-McGregor SM. Use of family care indicators and their relationship with child development in Bangladesh. J Heal Popul Nutr. 2010;28(1):23–33.
7. Jeong J, Pitchik HO, Yousafzai AK. Stimulation Interventions and Parenting in Low- and Middle-Income Countries: A Meta-analysis. Pediatrics. 2018;141(4):e20173510.
8. Frongillo EA, Kulkarni S, Basnet S, de Castro F. Family Care Behaviors and Early Childhood Development in Low- and Middle-Income Countries. J Child Fam Stud. 2017 Nov 13;26(11):3036–44.

Supplemental Table 1 Demographic and Health Surveys (DHS) included in the sample

**Country**	**Phase**	**Year**	**N**
Benin	Phase VII	2017-2018	2,029
Burundi	Phase VII	2016-2017	1,240
Cambodia	Phase VII	2014	385
Cameroon	Phase VI	2011	1,020
Chad	Phase VII	2014-2015	1,856
Congo	Phase VI	2011-2012	724
Haiti	Phase VII	2016-2017	427
Honduras	Phase VI	2011-2012	488
Jordan	Phase VII	2017-2018	766
Maldives	Phase VII	2016-2017	216
Rwanda	Phase VII	2014-2015	484
Senegal	Phase VII	2019	902
Timor-Leste	Phase-VII	2016	360
Togo	Phase VI	2013-2014	503
Uganda	Phase VII	2016	725

Bliznashka L, *et al. Arch Dis Child* 2022; 107:719–725. doi: 10.1136/archdischild-2021-323218

Supplemental Table 2 Mean age (in months) of children who can and children who cannot each milestone of the Early Childhood Development Index

Domain	Milestone	Can perform milestone	Cannot perform milestone
Cognitive	Follows simple directions on how to do something correctly	47.39±6.81	46.49±6.70
	When given something to do, is able to do it independently	47.52±6.78	46.45±6.76
Socio-emotional	Gets along well with other children	47.11±6.78	47.2±6.85
	Does not kick, bite, or hit other children	47.15±6.76	47.08±6.84
	Does not get distracted easily	46.86±6.79	47.33±6.78
Literacy-numeracy	Can identify/name at least ten letters of the alphabet	48.42±6.71	46.89±6.78
	Can read at least four simple, popular words	47.89±6.83	47.00±6.78
	Knows the name and recognizes the symbol of all numbers from 1 to 10	48.81±6.80	46.74±6.73
Physical	Can pick up a small object with two fingers, like a stick or a rock from the ground	47.27±6.80	46.41±6.72
	Is sometimes too sick to play	47.05±6.78	47.20±6.80

Bliznashka L, *et al. Arch Dis Child* 2022; 107:719–725. doi: 10.1136/archdischild-2021-323218

Supplemental Table 3 Multivariable adjusted associations between child dietary diversity score (range 0-7) and stimulation among children 36-59 months of age in 15 low- and middle-income countries[1]

	Number of stimulation activities received	Adequate stimulation received
	Adjusted Mean difference (95% CI)	Adjusted Relative risk (95% CI)
Benin	0.15 (0.10, 0.21)	1.08 (1.05, 1.11)
Burundi	0.07 (-0.00, 0.14)	1.03 (0.99, 1.07)
Cambodia	0.04 (-0.10, 0.18)	0.98 (0.89, 1.08)
Cameroon	0.02 (-0.05, 0.09)	1.00 (0.97, 1.03)
Chad	0.06 (-0.01, 0.14)	1.04 (1.00, 1.08)
Congo	0.06 (-0.08, 0.21)	1.05 (0.99, 1.11)
Haiti	0.04 (-0.09, 0.16)	1.03 (0.94, 1.12)
Honduras	0.11 (0.02, 0.20)	1.08 (1.01, 1.14)
Jordan	0.16 (0.07, 0.24)	1.04 (1.02, 1.07)
Maldives	0.04 (-0.00, 0.08)	1.01 (0.997, 1.03)
Rwanda	0.05 (-0.06, 0.17)	1.02 (0.95, 1.09)
Senegal	0.19 (0.10, 0.29)	1.10 (0.98, 1.23)
Timor-Leste	0.03 (-0.05, 0.12)	1.01 (0.98, 1.03)
Togo	-0.02 (-0.11, 0.07)	0.98 (0.89, 1.07)
Uganda	0.09 (-0.04, 0.22)	1.03 (0.98, 1.08)

[1] All models applied country-specific cluster variables and sampling weights. Estimates controlled for household wealth, rurality, size, access to improved sanitation, and access to improved water source; maternal age, education, and marital status; child age, sex, and attendance of an early childhood education programme.

Bliznashka L, *et al. Arch Dis Child* 2022; 107:719–725. doi: 10.1136/archdischild-2021-323218

Supplemental Table 4 Multivariable adjusted associations between child minimum dietary diversity (dietary diversity score ≥4) and stimulation among children 36-59 months of age in 15 low- and middle-income countries[1]

	Number of stimulation activities received<br>Adjusted<br>Mean difference<br>(95% CI)	Adequate stimulation received<br>Adjusted<br>Relative risk<br>(95% CI)
Benin	0.72 (0.45, 0.98)	1.45 (1.29, 1.64)
Burundi	0.42 (0.13, 0.70)	1.18 (1.03, 1.35)
Cambodia	0.25 (-0.26, 0.76)	1.08 (0.77, 1.52)
Cameroon	0.23 (-0.05, 0.51)	1.03 (0.89, 1.18)
Chad	0.41 (-0.04, 0.86)	1.24 (1.04, 1.49)
Congo	0.47 (-0.23, 1.17)	1.32 (1.04, 1.67)
Haiti	0.23 (-0.34, 0.80)	1.21 (0.84, 1.74)
Honduras	0.63 (0.26, 1.00)	1.43 (1.11, 1.85)
Jordan	0.48 (0.14, 0.82)	1.17 (1.08, 1.27)
Maldives	0.22 (0.05, 0.39)	1.05 (0.98, 1.11)
Rwanda	0.00 (-0.48, 0.48)	0.95 (0.71, 1.27)
Senegal	0.94 (0.45, 1.42)	1.43 (0.87, 2.35)
Timor-Leste	-0.14 (-0.62, 0.33)	0.92 (0.80, 1.05)
Togo	-0.20 (-0.60, 0.20)	0.53 (0.32, 0.87)
Uganda	0.32 (-0.20, 0.85)	1.14 (0.92, 1.41)

[1] All models applied country-specific cluster variables and sampling weights. Estimates controlled for household wealth, rurality, size, access to improved sanitation, and access to improved water source; maternal age, education, and marital status; child age, sex, and attendance of an early childhood education programme.

Bliznashka L, *et al. Arch Dis Child* 2022; 107:719–725. doi: 10.1136/archdischild-2021-323218

Supplemental Table 5 Multivariable adjusted associations between child consumption of animal sourced foods and stimulation among children 36-59 months of age in 15 low- and middle-income countries[1]

	Number of stimulation activities received<br>Adjusted Mean difference (95% CI)	Adequate stimulation received<br>Adjusted Relative risk (95% CI)
Benin	0.35 (0.13, 0.57)	1.20 (1.07, 1.36)
Burundi	0.17 (-0.10, 0.44)	1.06 (0.93, 1.20)
Cambodia	-0.08 (-0.54, 0.39)	0.83 (0.59, 1.16)
Cameroon	0.22 (-0.03, 0.48)	1.10 (0.96, 1.25)
Chad	0.14 (-0.10, 0.39)	1.09 (0.97, 1.22)
Congo	-0.07 (-0.49, 0.36)	0.96 (0.80, 1.15)
Haiti	0.27 (-0.23, 0.77)	1.07 (0.80, 1.43)
Honduras	0.41 (0.02, 0.80)	1.31 (0.99, 1.73)
Jordan	0.60 (0.26, 0.94)	1.19 (1.08, 1.30)
Maldives	0.15 (-0.04, 0.34)	1.06 (0.99, 1.13)
Rwanda	-0.15 (-0.66, 0.35)	0.85 (0.60, 1.20)
Senegal	0.53 (0.22, 0.84)	1.31 (0.91, 1.87)
Timor-Leste	0.29 (-0.08, 0.67)	1.07 (0.95, 1.20)
Togo	0.04 (-0.27, 0.35)	1.06 (0.77, 1.46)
Uganda	0.33 (-0.13, 0.79)	1.14 (0.95, 1.37)

[1] All models applied country-specific cluster variables and sampling weights. Estimates controlled for household wealth, rurality, size, access to improved sanitation, and access to improved water source; maternal age, education, and marital status; child age, sex, and attendance of an early childhood education programme.

Supplemental Table 6 Multivariable adjusted associations between child dietary diversity score (range 0-7) and child development among children 36-59 months of age in 15 low- and middle-income countries[1]

	Overall development off-track Adjusted Relative risk (95% CI)	Cognitive development off-track Adjusted Relative risk (95% CI)	Socio-emotional development off-track Adjusted Relative risk (95% CI)	Literacy-numeracy development off-track Adjusted Relative risk (95% CI)	Physical development off-track Adjusted Relative risk (95% CI)
Benin	1.07 (1.00, 1.14)	1.01 (0.96, 1.07)	1.04 (1.00, 1.08)	1.00 (0.99, 1.00)	1.05 (0.98, 1.12)
Burundi	0.95 (0.87, 1.03)	0.96 (0.91, 1.01)	1.02 (0.97, 1.07)	1.00 (0.98, 1.01)	0.98 (0.81, 1.18)
Cambodia	1.04 (0.81, 1.32)	0.93 (0.78, 1.10)	1.08 (0.95, 1.23)	1.03 (0.99, 1.07)	1.06 (0.87, 1.28)
Cameroon	1.03 (0.89, 1.18)	1.06 (0.96, 1.18)	0.95 (0.90, 0.99)	0.99 (0.98, 1.01)	0.93 (0.75, 1.15)
Chad	0.97 (0.90, 1.05)	0.98 (0.93, 1.03)	1.03 (0.98, 1.09)	0.99 (0.98, 1.00)	1.05 (0.96, 1.14)
Congo	1.07 (0.92, 1.24)	1.04 (0.90, 1.22)	0.95 (0.87, 1.03)	0.97 (0.95, 1.00)	1.24 (1.06, 1.44)
Haiti	1.08 (0.90, 1.30)	1.03 (0.87, 1.21)	1.06 (0.96, 1.18)	1.01 (0.98, 1.03)	1.18 (0.94, 1.49)
Honduras	1.23 (0.96, 1.57)	0.98 (0.80, 1.19)	1.00 (0.93, 1.07)	1.00 (0.99, 1.02)	0.96 (0.76, 1.22)
Jordan	0.87 (0.79, 0.96)	0.88 (0.80, 0.97)	1.04 (0.98, 1.11)	1.02 (0.98, 1.06)	0.94 (0.84, 1.04)
Maldives	0.21 (0.17, 0.26)	0.95 (0.77, 1.18)	0.99 (0.90, 1.09)	0.89 (0.77, 1.02)	0.00 (0.00, 0.00)
Rwanda	1.04 (0.79, 1.39)	1.06 (0.89, 1.26)	1.09 (0.96, 1.23)	1.01 (0.99, 1.02)	0.78 (0.61, 1.00)
Senegal	1.02 (0.88, 1.19)	1.18 (1.05, 1.34)	0.97 (0.88, 1.07)	1.00 (0.996, 1.01)	0.94 (0.81, 1.11)
Timor-Leste	0.94 (0.85, 1.03)	0.98 (0.91, 1.05)	1.08 (0.99, 1.17)	0.94 (0.89, 1.00)	0.92 (0.84, 1.01)
Togo	0.82 (0.66, 1.01)	0.90 (0.81, 1.00)	1.03 (0.93, 1.15)	1.01 (0.99, 1.02)	0.77 (0.57, 1.04)
Uganda	0.97 (0.79, 1.20)	1.10 (0.94, 1.28)	1.05 (0.96, 1.14)	0.97 (0.94, 1.00)	0.84 (0.68, 1.04)

[1] All models applied country-specific cluster variables and sampling weights. Estimates controlled for household wealth, rurality, size, access to improved sanitation, and access to improved water source; maternal age, education, and marital status; child age, sex, and attendance of an early childhood education programme.

Supplemental Table 7 Multivariable adjusted associations between child minimum dietary diversity (dietary diversity score ≥4) and child development among children 36-59 months of age in 15 low- and middle-income countries[1]

	Overall development off-track Adjusted Relative risk (95% CI)	Cognitive development off-track Adjusted Relative risk (95% CI)	Socio-emotional development off-track Adjusted Relative risk (95% CI)	Literacy-numeracy development off-track Adjusted Relative risk (95% CI)	Physical development off-track Adjusted Relative risk (95% CI)
Benin	1.44 (1.10, 1.89)	1.09 (0.86, 1.38)	1.13 (0.93, 1.38)	0.98 (0.94, 1.02)	1.48 (1.14, 1.91)
Burundi	0.82 (0.55, 1.22)	0.72 (0.54, 0.96)	1.10 (0.90, 1.34)	0.98 (0.91, 1.06)	1.56 (0.86, 2.82)
Cambodia	0.60 (0.17, 2.07)	0.59 (0.22, 1.53)	1.37 (0.85, 2.20)	1.00 (0.85, 1.17)	0.54 (0.18, 1.59)
Cameroon	1.08 (0.62, 1.88)	1.30 (0.89, 1.92)	0.70 (0.55, 0.88)	1.00 (0.93, 1.07)	0.76 (0.27, 2.14)
Chad	0.77 (0.49, 1.22)	0.75 (0.54, 1.04)	1.30 (0.97, 1.73)	0.98 (0.91, 1.04)	1.13 (0.69, 1.83)
Congo	0.58 (0.19, 1.72)	1.33 (0.60, 2.93)	0.71 (0.48, 1.05)	0.92 (0.78, 1.09)	1.20 (0.42, 3.44)
Haiti	1.42 (0.64, 3.15)	0.98 (0.42, 2.29)	1.09 (0.69, 1.70)	0.98 (0.86, 1.11)	1.75 (0.64, 4.77)
Honduras	2.39 (1.03, 5.58)	1.02 (0.45, 2.35)	0.99 (0.73, 1.36)	1.01 (0.96, 1.07)	0.90 (0.38, 2.17)
Jordan	0.48 (0.25, 0.91)	0.57 (0.32, 1.02)	1.23 (0.92, 1.64)	1.01 (0.84, 1.22)	0.62 (0.35, 1.08)
Maldives	0.00 (0.00, 0.00)	0.64 (0.18, 2.22)	1.23 (0.78, 1.94)	0.56 (0.25, 1.24)	0.00 (0.00, 0.00)
Rwanda	1.73 (0.65, 4.63)	0.97 (0.44, 2.13)	1.43 (0.87, 2.37)	1.05 (0.99, 1.12)	0.46 (0.15, 1.46)
Senegal	0.25 (0.07, 0.88)	1.04 (0.43, 2.52)	0.86 (0.50, 1.49)	1.00 (0.98, 1.02)	0.16 (0.04, 0.74)
Timor-Leste	0.7 (0.42, 1.18)	0.93 (0.65, 1.32)	1.14 (0.75, 1.75)	0.83 (0.65, 1.06)	0.63 (0.38, 1.03)
Togo	1.20 (0.51, 2.81)	0.91 (0.53, 1.55)	1.38 (0.85, 2.23)	1.04 (0.97, 1.11)	0.56 (0.15, 2.13)
Uganda	0.76 (0.27, 2.10)	1.14 (0.62, 2.08)	1.29 (0.89, 1.86)	0.87 (0.75, 1.00)	0.54 (0.20, 1.48)

[1] All models applied country-specific cluster variables and sampling weights. Estimates controlled for household wealth, rurality, size, access to improved sanitation, and access to improved water source; maternal age, education, and marital status; child age, sex, and attendance of an early childhood education programme.

Supplemental Table 8 Multivariable adjusted associations between child consumption of animal sourced foods and child development among children 36-59 months of age in 15 low- and middle-income countries[1]

	Overall development off-track Adjusted Relative risk (95% CI)	Cognitive development off-track Adjusted Relative risk (95% CI)	Socio-emotional development off-track Adjusted Relative risk (95% CI)	Literacy-numeracy development off-track Adjusted Relative risk (95% CI)	Physical development off-track Adjusted Relative risk (95% CI)
Benin	1.25 (0.99, 1.59)	1.13 (0.93, 1.37)	0.98 (0.84, 1.16)	1.00 (0.98, 1.03)	1.11 (0.88, 1.42)
Burundi	0.86 (0.61, 1.21)	0.75 (0.59, 0.97)	1.12 (0.93, 1.35)	0.98 (0.92, 1.04)	1.50 (0.81, 2.77)
Cambodia	0.80 (0.27, 2.40)	0.92 (0.46, 1.83)	1.30 (0.80, 2.12)	1.09 (0.95, 1.24)	0.99 (0.44, 2.24)
Cameroon	1.13 (0.73, 1.76)	1.18 (0.86, 1.62)	0.88 (0.74, 1.03)	1.00 (0.95, 1.04)	0.74 (0.33, 1.62)
Chad	0.85 (0.68, 1.06)	0.99 (0.85, 1.14)	1.01 (0.84, 1.20)	0.98 (0.94, 1.01)	1.13 (0.86, 1.48)
Congo	1.48 (0.87, 2.50)	1.14 (0.76, 1.71)	0.95 (0.75, 1.20)	0.92 (0.86, 0.99)	1.87 (1.04, 3.37)
Haiti	0.86 (0.45, 1.65)	0.67 (0.35, 1.26)	1.36 (0.97, 1.91)	0.97 (0.89, 1.07)	1.48 (0.79, 2.79)
Honduras	1.59 (0.55, 4.61)	0.79 (0.38, 1.62)	1.25 (0.90, 1.75)	0.98 (0.92, 1.03)	3.30 (0.60, 18.20)
Jordan	0.80 (0.48, 1.31)	0.92 (0.58, 1.45)	1.20 (0.89, 1.62)	1.12 (0.94, 1.33)	1.06 (0.69, 1.64)
Maldives	0.00 (0.00, 0.00)	0.81 (0.28, 2.36)	1.29 (0.83, 2.01)	0.56 (0.24, 1.29)	0.00 (0.00, 0.00)
Rwanda	0.88 (0.21, 3.74)	0.77 (0.31, 1.89)	1.81 (1.11, 2.97)	1.04 (0.98, 1.11)	0.32 (0.07, 1.53)
Senegal	1.66 (1.01, 2.71)	1.58 (1.04, 2.40)	1.02 (0.76, 1.39)	1.00 (0.98, 1.01)	1.08 (0.61, 1.92)
Timor-Leste	0.77 (0.52, 1.15)	1.01 (0.76, 1.35)	1.64 (1.10, 2.44)	0.87 (0.71, 1.07)	0.70 (0.46, 1.05)
Togo	0.54 (0.30, 1.00)	0.81 (0.57, 1.15)	1.00 (0.69, 1.44)	1.01 (0.96, 1.06)	0.29 (0.12, 0.70)
Uganda	0.76 (0.35, 1.67)	1.16 (0.71, 1.88)	1.19 (0.89, 1.59)	0.92 (0.83, 1.03)	0.64 (0.29, 1.44)

[1] All models applied country-specific cluster variables and sampling weights. Estimates controlled for household wealth, rurality, size, access to improved sanitation, and access to improved water source; maternal age, education, and marital status; child age, sex, and attendance of an early childhood education programme.

Bliznashka L, *et al. Arch Dis Child* 2022; 107:719–725. doi: 10.1136/archdischild-2021-323218

Supplemental Table 9 Associations between child diet and child development among children 36-59 months of age in 15 low- and middle-income countries[1]

	Early Childhood Development Index	
	Unadjusted Mean difference (95% CI)	Adjusted Mean difference (95% CI)
Dietary diversity score (0-7)	0.13 (0.10, 0.15)	0.02 (-0.00, 0.04)
Minimum dietary diversity (≥4 food groups)	0.60 (0.48, 0.72)	0.12 (0.01, 0.23)
Consumed animal source foods	0.28 (0.20, 0.37)	0.02 (-0.06, 0.11)

[1] All models applied country-specific cluster variables and sampling weights. Adjusted estimates controlled for household wealth, rurality, size, access to improved sanitation, and access to improved water source; maternal age, education, and marital status; stimulation; child age, sex, and attendance of an early childhood education programme; and country and survey year

Bliznashka L, *et al. Arch Dis Child* 2022; 107:719–725. doi: 10.1136/archdischild-2021-323218

Supplemental Table 10 Heterogeneity of the association between child minimum dietary diversity and child development by child, maternal, and household factors, comparing children who met minimum dietary diveristy to children who did not[1]

	**Overall development off-track**	**Cognitive development off-track**	**Socio-emotional development off-track**	**Literacy-numeracy development off-track**	**Physical development off-track**
Child sex					
Girl	0.87 (0.68, 1.10)	0.88 (0.74, 1.05)	0.99 (0.87, 1.13)	0.98 (0.95, 1.02)	0.81 (0.62, 1.07)
Boy	0.97 (0.78, 1.21)	0.93 (0.78, 1.10)	1.08 (0.96, 1.21)	0.96 (0.92, 1.00)	1.12 (0.88, 1.42)
p-value for interaction	0.50	0.65	0.33	0.32	0.07
Child age					
36-48 mo	0.88 (0.71, 1.10)	0.87 (0.75, 1.02)	1.10 (0.97, 1.24)	0.98 (0.94, 1.01)	0.91 (0.71, 1.16)
48-59 mo	0.99 (0.78, 1.25)	0.96 (0.80, 1.14)	0.98 (0.87, 1.11)	0.97 (0.93, 1.00)	1.10 (0.85, 1.42)
p-value for interaction	0.45	0.43	0.18	0.69	0.26
Adequate stimulation					
No	1.00 (0.78, 1.28)	1.06 (0.88, 1.27)	0.96 (0.83, 1.11)	1.02 (1.00, 1.04)	1.15 (0.90, 1.47)
Yes	0.87 (0.69, 1.10)	0.80 (0.67, 0.95)	1.09 (0.97, 1.22)	0.93 (0.89, 0.97)	0.86 (0.66, 1.11)
p-value for interaction	0.42	0.03	0.17	<0.01	0.09
Child attends an early childhood care and education programme					
No	0.90 (0.76, 1.08)	0.87 (0.77, 0.99)	1.01 (0.91, 1.11)	0.99 (0.97, 1.02)	0.98 (0.80, 1.20)
Yes	1.28 (0.79, 2.09)	1.35 (0.94, 1.95)	1.15 (0.95, 1.38)	0.87 (0.79, 0.97)	1.04 (0.56, 1.93)
p-value for interaction	0.18	0.02	0.21	0.02	0.86
Maternal education					
None	1.04 (0.84, 1.29)	0.89 (0.75, 1.06)	1.15 (0.99, 1.32)	1.02 (1.00, 1.04)	1.23 (0.97, 1.56)
Primary	0.92 (0.66, 1.28)	1.00 (0.80, 1.26)	0.98 (0.84, 1.15)	0.98 (0.95, 1.02)	0.84 (0.58, 1.23)
Secondary or higher	0.75 (0.51, 1.09)	0.84 (0.63, 1.11)	0.99 (0.84, 1.17)	0.88 (0.81, 0.97)	0.77 (0.53, 1.11)
p-value for interaction	0.34	0.58	0.30	<0.01	0.05
Maternal age					
15-24 y	1.17 (0.87, 1.58)	1.09 (0.85, 1.39)	0.90 (0.73, 1.11)	1.02 (0.97, 1.06)	1.15 (0.85, 1.56)
24-39 y	0.87 (0.71, 1.07)	0.88 (0.75, 1.02)	1.09 (0.98, 1.21)	0.95 (0.92, 0.98)	0.90 (0.71, 1.13)
40-49 y	0.69 (0.36, 1.34)	0.65 (0.39, 1.08)	0.92 (0.67, 1.26)	1.07 (0.98, 1.17)	1.24 (0.62, 2.47)
p-value for interaction	0.17	0.16	0.21	<0.01	0.32

Bliznashka L, *et al. Arch Dis Child* 2022; 107:719–725. doi: 10.1136/archdischild-2021-323218

Married or cohabitating					
No	1.78 (0.88, 3.57)	1.28 (0.73, 2.26)	0.76 (0.47, 1.20)	1.02 (0.92, 1.13)	1.67 (0.75, 3.69)
Yes	0.91 (0.76, 1.07)	0.90 (0.79, 1.02)	1.05 (0.96, 1.15)	0.97 (0.94, 1.00)	0.97 (0.80, 1.17)
p-value for interaction	0.06	0.23	0.17	0.33	0.18
Household wealth quintile					
Poorest	0.94 (0.70, 1.27)	0.87 (0.69, 1.10)	1.00 (0.84, 1.20)	1.00 (0.96, 1.04)	1.11 (0.82, 1.50)
Poorer	1.25 (0.88, 1.76)	1.05 (0.80, 1.37)	1.09 (0.91, 1.32)	0.99 (0.94, 1.05)	1.27 (0.89, 1.81)
Middle	0.94 (0.68, 1.31)	1.06 (0.82, 1.36)	1.01 (0.84, 1.23)	0.98 (0.93, 1.04)	0.91 (0.62, 1.33)
Richer	0.65 (0.44, 0.96)	0.61 (0.45, 0.82)	1.02 (0.84, 1.25)	0.99 (0.94, 1.05)	0.94 (0.63, 1.41)
Richest	0.89 (0.54, 1.46)	1.00 (0.70, 1.44)	1.07 (0.84, 1.35)	0.87 (0.79, 0.95)	0.55 (0.30, 1.00)
p-value for interaction	0.20	0.04	0.97	0.10	0.13
Household location					
Urban	0.82 (0.58, 1.15)	0.96 (0.75, 1.23)	1.02 (0.87, 1.20)	0.94 (0.88, 1.00)	0.77 (0.54, 1.10)
Rural	0.98 (0.81, 1.18)	0.88 (0.77, 1.02)	1.05 (0.95, 1.16)	0.99 (0.96, 1.01)	1.12 (0.92, 1.37)
p-value for interaction	0.38	0.55	0.79	0.14	0.06
Household has improved water					
No	1.13 (0.84, 1.53)	1.07 (0.86, 1.34)	1.02 (0.85, 1.21)	1.01 (0.97, 1.05)	1.40 (1.07, 1.85)
Yes	0.87 (0.70, 1.07)	0.86 (0.74, 1.00)	1.04 (0.93, 1.15)	0.96 (0.93, 1.00)	0.90 (0.71, 1.14)
p-value for interaction	0.16	0.11	0.84	0.09	0.01
Household has improved sanitation					
No	1.06 (0.88, 1.27)	0.98 (0.85, 1.12)	1.03 (0.93, 1.15)	0.98 (0.96, 1.01)	1.23 (1.01, 1.51)
Yes	0.64 (0.44, 0.92)	0.74 (0.57, 0.97)	1.05 (0.90, 1.22)	0.94 (0.88, 1.01)	0.58 (0.40, 0.84)
p-value for interaction	0.01	0.07	0.83	0.24	<0.01

[1] Values are relative risk and 95% confidence intervals. All models applied country-specific cluster variables and sampling weights. Estimates controlled for household wealth, rurality, size, access to improved sanitation, and access to improved water source; maternal age, education, and marital status; stimulation; child age, sex, and attendance of an early childhood education programme; and country and survey year.

Supplemental Table 11 Heterogeneity of the association between child consumption of animal source foods and child development by pre-specified child, maternal, and household factors, comparing children who consumed animal source foods and children who did not[1]

	**Overall development off-track**	**Cognitive development off-track**	**Socio-emotional development off-track**	**Literacy-numeracy development off-track**	**Physical development off-track**
Child sex					
Girl	0.93 (0.78, 1.10)	1.02 (0.91, 1.15)	1.08 (0.97, 1.19)	1.00 (0.97, 1.02)	0.89 (0.72, 1.09)
Boy	1.01 (0.87, 1.18)	0.99 (0.89, 1.11)	1.02 (0.94, 1.12)	0.98 (0.96, 1.00)	1.15 (0.96, 1.37)
p-value for interaction	0.45	0.77	0.44	0.26	0.047
Child age					
36-48 mo	0.94 (0.81, 1.10)	0.97 (0.88, 1.08)	1.07 (0.98, 1.18)	1.00 (0.98, 1.02)	1.03 (0.86, 1.22)
48-59 mo	1.02 (0.86, 1.20)	1.05 (0.94, 1.19)	1.02 (0.93, 1.12)	0.98 (0.95, 1.00)	1.02 (0.83, 1.25)
p-value for interaction	0.51	0.32	0.43	0.21	0.94
Adequate stimulation					
No	0.96 (0.82, 1.13)	1.01 (0.9, 1.14)	0.97 (0.88, 1.07)	1.00 (0.99, 1.02)	1.04 (0.86, 1.25)
Yes	0.99 (0.83, 1.10)	1.00 (0.89, 1.12)	1.12 (1.01, 1.23)	0.97 (0.94, 1.00)	1.00 (0.82, 1.21)
p-value for interaction	0.81	0.84	0.04	0.03	0.75
Child attends an early childhood care and education programme					
No	0.97 (0.86, 1.10)	1.00 (0.92, 1.08)	1.03 (0.95, 1.11)	0.99 (0.97, 1.01)	1.02 (0.88, 1.18)
Yes	1.12 (0.73, 1.73)	1.19 (0.86, 1.64)	1.14 (0.97, 1.33)	0.96 (0.88, 1.04)	1.16 (0.69, 1.95)
p-value for interaction	0.52	0.30	0.26	0.40	0.63
Maternal education					
None	0.98 (0.83, 1.15)	0.97 (0.86, 1.08)	1.03 (0.92, 1.16)	1.00 (0.98, 1.02)	1.08 (0.90, 1.29)
Primary	1.01 (0.81, 1.27)	1.08 (0.93, 1.26)	1.06 (0.95, 1.18)	1.00 (0.97, 1.02)	0.98 (0.74, 1.31)
Secondary or higher	0.90 (0.67, 1.22)	0.99 (0.79, 1.25)	1.05 (0.91, 1.21)	0.95 (0.89, 1.01)	0.94 (0.7, 1.27)
p-value for interaction	0.84	0.52	0.96	0.25	0.69
Maternal age					
15-24 y	1.06 (0.84, 1.33)	1.03 (0.87, 1.21)	1.02 (0.88, 1.19)	1.02 (0.99, 1.05)	1.17 (0.91, 1.50)
24-39 y	0.95 (0.82, 1.09)	1.00 (0.91, 1.10)	1.07 (0.99, 1.16)	0.98 (0.96, 1.00)	0.94 (0.79, 1.13)
40-49 y	0.94 (0.59, 1.48)	1.00 (0.73, 1.38)	0.84 (0.64, 1.09)	0.99 (0.94, 1.04)	1.34 (0.8, 2.22)
p-value for interaction	0.71	0.95	0.19	0.13	0.24

Bliznashka L, *et al. Arch Dis Child* 2022; 107:719–725. doi: 10.1136/archdischild-2021-323218

Married or cohabitating					
No	0.94 (0.46, 1.89)	0.93 (0.56, 1.56)	0.74 (0.51, 1.07)	0.97 (0.90, 1.06)	0.86 (0.40, 1.83)
Yes	0.97 (0.87, 1.10)	1.01 (0.93, 1.09)	1.06 (0.99, 1.14)	0.99 (0.97, 1.01)	1.03 (0.89, 1.19)
p-value for interaction	0.91	0.77	0.06	0.71	0.65
Household wealth quintile					
Poorest	1.14 (0.94, 1.37)	1.06 (0.94, 1.20)	1.07 (0.95, 1.20)	1.00 (0.98, 1.03)	1.17 (0.94, 1.47)
Poorer	0.97 (0.75, 1.25)	1.07 (0.89, 1.27)	1.01 (0.87, 1.18)	1.01 (0.98, 1.04)	1.05 (0.8, 1.39)
Middle	0.85 (0.67, 1.09)	0.95 (0.78, 1.15)	0.98 (0.85, 1.13)	1.01 (0.97, 1.05)	1.00 (0.76, 1.30)
Richer	0.96 (0.75, 1.25)	0.91 (0.75, 1.10)	1.11 (0.95, 1.30)	0.99 (0.95, 1.03)	1.05 (0.76, 1.46)
Richest	0.85 (0.57, 1.29)	1.00 (0.75, 1.35)	1.08 (0.87, 1.32)	0.87 (0.81, 0.94)	0.63 (0.39, 1.01)
p-value for interaction	0.38	0.61	0.79	0.01	0.23
Household location					
Urban	1.03 (0.79, 1.35)	1.03 (0.85, 1.25)	1.07 (0.93, 1.23)	0.95 (0.91, 1.00)	0.93 (0.69, 1.25)
Rural	0.95 (0.84, 1.08)	1.00 (0.91, 1.09)	1.03 (0.96, 1.12)	1.00 (0.99, 1.02)	1.06 (0.91, 1.24)
p-value for interaction	0.57	0.73	0.66	0.03	0.41
Household has improved water					
No	1.01 (0.84, 1.21)	1.11 (0.98, 1.25)	0.95 (0.84, 1.07)	1.01 (0.98, 1.03)	1.20 (0.97, 1.47)
Yes	0.96 (0.82, 1.12)	0.96 (0.86, 1.06)	1.08 (0.99, 1.18)	0.97 (0.95, 1.00)	0.95 (0.79, 1.14)
p-value for interaction	0.70	0.07	0.08	0.055	0.09
Household has improved sanitation					
No	1.00 (0.87, 1.14)	1.02 (0.93, 1.12)	1.02 (0.94, 1.11)	0.99 (0.97, 1.01)	1.06 (0.90, 1.24)
Yes	0.89 (0.68, 1.17)	0.94 (0.78, 1.13)	1.12 (0.99, 1.27)	0.99 (0.94, 1.03)	0.92 (0.70, 1.22)
p-value for interaction	0.48	0.44	0.20	0.97	0.38

[1] Values are relative risk and 95% confidence intervals. All models applied country-specific cluster variables and sampling weights. Estimates controlled for household wealth, rurality, size, access to improved sanitation, and access to improved water source; maternal age, education, and marital status; stimulation; child age, sex, and attendance of an early childhood education programme; and country and survey year.
